# RAGE regulates oxytocin transport into the brain

**DOI:** 10.1038/s42003-020-0799-2

**Published:** 2020-02-13

**Authors:** Yasuhiko Yamamoto, Haruhiro Higashida

**Affiliations:** 1 Departments of Biochemistry and Molecular Vascular Biology, Kanazawa University Graduate School of Medical Sciences, Kanazawa, 920-8640 Japan; 2 Department of Basic Research on Social Recognition and Memory, Research Centre for Child Mental Development, Kanazawa University, Kanazawa, 920-8640 Japan

## Abstract

Oxytocin, a nonapeptide hormone, has a key role in female reproductive functions as well as in social memory in the brain. In our recent *Communications Biology* article, we reported that oxytocin is transported from the peripheral blood into the brain by the receptor for advanced glycation end-products (RAGE) in endothelial cells at the blood−brain barrier. Additionally, we found that oral oxytocin is absorbed by RAGE on intestinal epithelial cells at the blood−intestinal barrier. From a physiological perspective, we herein outline the continuing research regarding oxytocin and social behaviour.

## Abstract (web summary)

Yamamoto and Higashido discuss the possible routes of the hormone oxytocin in the body, and highlight their recent study in *Communications Biology* where they showed that the RAGE receptor is a transporter for oxytocin across the blood−brain barrier.

## Oxytocin in female reproduction and beyond

Oxytocin is primarily synthesized in the oxytocinergic neurons in the hypothalamus within the brain^1,2^. Oxytocin in brain neurons is secreted somato-axono-dendritically into the brain^3,4^ and released from the nerve terminals in the posterior pituitary into the circulation^1–4^. Within the past decade, oxytocin in the brain has attracted increasing attention because of the importance of oxytocin in species-specific social memory functioning^5–7^. Oxytocin in the blood circulation has a primary hormonal role in female reproduction but was not thought to re-enter the brain. Recently, however, based on the practical nasal administration of large doses of oxytocin to humans with and without social deficit-related psychiatric disorders, such as autism spectrum disorders and schizophrenia^8,9^, oxytocin has been thought to cross the blood−brain barrier (BBB)^3,10^. However, there is little or no direct evidence for this transport process, with the exception of two reports that examined human cerebrospinal fluid (CSF) and microdialysates from the mouse and rat hippocampus and amygdala^11,12^. They found that peak levels of oxytocin were observed 30–60 min after the nasal administration of oxytocin in three species^12,13^, which is confirmed in our study as well^14^. Furthermore, if oxytocin is indeed transferred from the blood to the brain, the underlying molecular mechanism is unclear.

## How oxytocin crosses the blood−brain barrier

The transport of peptides across the BBB requires specific or nonspecific interactions with proteins or receptors that are expressed on the luminal and/or abluminal surfaces of brain capillary cells^15^. Endocytosis and transcytosis play important roles in uni- and bidirectional transport across the BBB. Therefore, it was necessary to hypothesize that oxytocin transfer across the BBB is mediated by a binding protein^15^. We considered that such a protein should meet the following criteria: it should exist on brain microvessel endothelial cells; it should have multiligand binding capacity (non-oxytocin-specific receptors exist, for example in the uterus); and its binding to oxytocin should not be popularly known.

Regarding possible candidates, we suspected the receptor for advanced glycation end-products (receptor for AGEs, RAGE), which belongs to the immunoglobulin superfamily and is involved in a variety of diseases related to inflammation and blood vessel impediments caused by diabetes^16–22^. Membrane-bound full-length RAGE (mRAGE) is expressed on the cell surfaces of the endothelial cells of various organs, including the endothelial cells of neurovascular units^23^. Additionally, plasma contains the soluble form of RAGE (sRAGE), which is composed of endogenous soluble RAGE (esRAGE), an alternative splicing product of the *AGER* gene and an ectodomain-shedded form of mRAGE^20^. These different forms of RAGE possess identical multiple ligand binding properties for AGE, high mobility group box 1 (HMGB1), S100 proteins, lipopolysaccharides, and soluble amyloid-beta peptide^18,19^ but have never been demonstrated to bind to physiological ligands, such as oxytocin. The interaction of β-amyloid and RAGE results in its transport across the BBB^23^. Thus, RAGE seems to fulfill the aforementioned criteria, and we hypothesized that RAGE is a candidate protein for the mediation of oxytocin transfer from the blood to the brain.

**Fig. 1:**
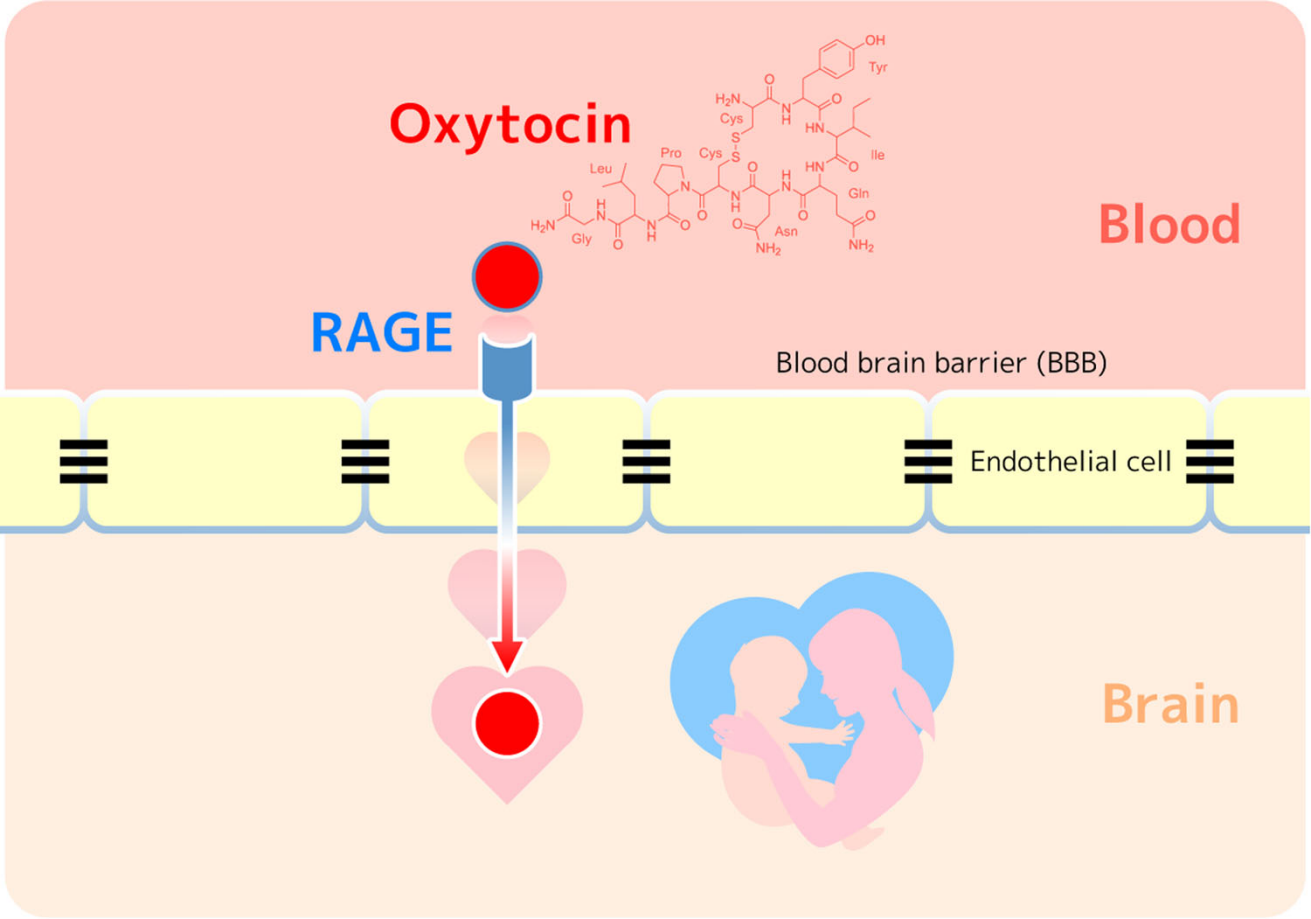
RAGE-dependent oxytocin transport from the blood into the brain via the blood−brain barrier (BBB). Oxytocin is a peptide hormone released by the posterior pituitary and plays important roles in maternal behaviors and social bonding. Endothelial RAGE is an essential molecule existing at the BBB for the oxytocin transfer and the functions.

Indeed, we demonstrated that RAGE is the oxytocin’s binding protein using the plasmon resonance method and that it is a transporter of oxytocin primarily from the blood to the brain using reconstituted BBB in culture^14^. Interestingly, the transfer is rather unidirectional; the blood to brain direction is 5–10-fold more efficient than transfer in the reverse direction (brain to blood). It has only recently been proposed that blood oxytocin re-enters the brain (Fig. 1)^14^.

## Possible routes and functions of oxytocin

Although oxytocin receptors have been identified in numerous brain regions^24^ and occasionally in remote brain areas far from the oxytocin synthesizing neurons in the hypothalamus^2,24^, oxytocin transmission is believed to occur either via release from collateral axons into the amygdala or nucleus accumbens or via diffusion processes through extracellular spaces in a process known as volume transmission^2^. Interestingly, our finding regarding RAGE suggests a third possibility, in that oxytocin is able to function in brain after transport from the blood (Fig. 1)^14^. This system might allow the transfer of oxytocin’s peripheral information to the central nervous system during labor, lactation, or social interactions with or without stress.

More characteristically, the binding of oxytocin as a ligand to RAGE did not induce the activation of intracellular signaling, which contrasts with the binding of AGEs, S100, HMGB1, and amyloid β^14^. The lack of activation of cellular responses by oxytocin strongly supports the role of RAGE over other cell remodeling activities in pathological conditions such as diabetes, renal or retinal vessel dysfunction, and Alzheimer’s disease^16–23^.

The presence of RAGE in the caveolae of the hippocampus and choroid plexus suggests that RAGE might be a carrier of oxytocin in vesicles (endocytosis) or for transcytosis in a manner similar to that suggested for Mfsd2a and omega-3 fatty acids^25^. However, it is not clear at the cellular level how RAGE transports oxytocin into the brain.

RAGE seems to play a role not only in the BBB, but also in the blood−CSF barrier because RAGE is found in the choroid plexus^14^. The transport of oxytocin to the CSF might thus be mediated via the choroid plexus, which could be one of the reasons that the intranasal administration of oxytocin is effective in the central delivery of oxytocin and results in beneficial effects on impaired social interaction behavior^6,8^. We suggest that the circulation route for the rapid transfer of oxytocin following intranasal administration is via a specific nasal structure and that it passes through intercellular clefts in the olfactory epithelium to diffuse into the subarachnoid space thus reaching the choroid plexus^26^. However, even if this is the case, RAGE might play an important role.

In human neurovascular units, RAGE is confirmed to be present in vascular endothelial cells in the human brain^27^. Therefore, since the physiological properties of RAGE do not differ between human and mice, it is reasonable to hypothesize that RAGE contributes to the transport of plasma oxytocin to the brain in humans. Further, oxytocin transfer to the brain is increased when RAGE expression is enhanced by transient brain ischemia^14^. This suggests that oxytocin plays an important role in the wound-healing process and/or stress after brain ischemic attack

Human plasma oxytocin has an affinity to esRAGE in the affinity column^14^. This suggests that esRAGE may be a binding protein in the circulation. When human plasma is pretreated to remove plasma proteins, the remaining concentration of free oxytocin in the plasma is estimated to be extremely low (<10 pg/ml, below the detectable level), suggesting that oxytocin is present in a protein-binding form. Although such binding proteins have not yet been identified in human plasma, it is highly possible that this protein could be esRAGE. This point should be further examined. Interestingly, it is also found that circulating esRAGE is transported into the brain through the BBB by mRAGE on endothelial cells, potentially contributing to the oxytocin transfer into the brain^28^.

RAGE-mediated oxytocin transport from the intestinal tract into the blood circulation across the intestinal barrier was also found to occur in mice^29^. This RAGE-dependent intestinal permeability was demonstrated following the oral administration of oxytocin in mouse pups at the very narrow window of postnatal days 4–6, during which the barrier will be formed but still digestion is not active. The blood oxytocin levels of the mice were high at approximately 1–3 days after birth, likely due to nonspecific bulk absorption, and no uptake of oxytocin was observed after 7 days when the same concentration of oxytocin was orally applied^29^. Although it was previously reported that oxytocin is absorbed in neonates and infants, our results show that oxytocin transport across the intestinal barrier is RAGE-dependent^29^.

Oxytocin absorption via the intestine was more difficult to detect in adults. However, when we used a tenfold higher concentration for gavage, the uptake was visible^29^. Oxytocin seems to be somewhat resistant to digestion. This result suggests that novel delivery methods for oral oxytocin supplementation may be advantageous for the treatment of a range of conditions, such as impaired social development in preterm infants who are at risk of autism spectrum disorder, and obesity or diabetes in adults^19^.

## Outlook

Future directions for currently unsolved questions include answering questions about whether oxytocin transport is increased in the excess of sRAGE or esRAGE via mRAGE. We would also like to know what extent does RAGE-dependent oxytocin transport rely on other transport means, such as the direct intranasal pathway or trigeminal nerve transport^26^. It will be important to follow up results on additional oxytocin binding proteins in plasma. The molecular weight of sRAGE/esRAGE is approximately 50 kDa. However, it is known that there is an oxytocin binding protein with a molecular weight of 10 kDa^30^. It has frequently been demonstrated that no oxytocin transport to the brain occurs in rats and it would be interesting to know if this could be due to lack of RAGE function. Another question is whether oxytocin recruited in cortical regions that are far from the hypothalamus with no axon collaterals from oxytocinergic neurons^2^. With regards to behavior, there is one hypothesis that personal differences in social behavior in humans depends on plasma oxytocin levels. Given our data, it is reasonable to ask whether molecular variants or single nucleotide polymorphisms of the *AGER* gene coding RAGE contribute to this phenomenon. Further, can orally administered oxytocin reach the brain? This is particularly relevant for oxytocin in milk. We also need to determine the precise bioavailability of oxytocin from the intestine and the blood. Several questions arise about oxytocin movement. Can oxytocin enter the lymph and intraperitoneal space, and tight junctions? Can oxytocin be absorbed from mouse epithelial cells and esophageal epithelial cells in a RAGE-dependent fashion across epithelial barriers? Overall, it will be important to determine how oxytocin is transported intracellularly, transcellularly and intercellularly^25^.

To summarize, although we need to determine the pharmacokinetics of oxytocin, our data present a better understanding of how exogenous oxytocin from the intestine or blood can influence the brain targets of oxytocin, which results in social behavioral improvement in humans with or without social impairments.
